# Identifying the metabolic perturbations in earthworm induced by cypermethrin using gas chromatography-mass spectrometry based metabolomics

**DOI:** 10.1038/srep15674

**Published:** 2015-10-30

**Authors:** Ratnasekhar Ch, Amit Kumar Singh, Pathya Pandey, Prem Narain Saxena, Mohana Krishna Reddy Mudiam

**Affiliations:** 1 Analytical Chemistry Laboratory & Regulatory Toxicology Group, CSIR- Indian Institute of Toxicology Research, P.O. Box 80, M.G. Marg, Lucknow-226001, Uttar Pradesh, India; 2 Academy of Scientific and Innovative Research, CSIR- IITR Main Campus, P.O. Box 80, M.G. Marg, Lucknow-226001, Uttar Pradesh, India; 3 SEM facility, CSIR- Indian Institute of Toxicology Research, P.O. Box 80, M.G. Marg, Lucknow-226001, Uttar Pradesh, India; 4 Pesticide Toxicology Laboratory & Regulatory Toxicology Group, CSIR- Indian Institute of Toxicology Research, P.O. Box 80, M.G. Marg, Lucknow-226001, Uttar Pradesh, India

## Abstract

Globally, cypermethrin is one of the most widely used synthetic pyrethroid for agricultural and domestic purposes. Most part of the pesticides used in the agriculture ends up as residues in the soil, making soil dwelling organisms, especially earthworms more susceptible to pesticide intoxication. Cypermethrin is known to be a neurotoxicant to many model organisms, including mammals and insects, but such type of toxicity evidence is not available for invertebrate systems like earthworms. In the present work, metabolomics based approach was utilized to identify the toxic mechanism of action of cypermethrin on earthworm (*Metaphire posthuma*) and these were exposed to sub-lethal concentrations of cypermethrin such as 2.5 mg/kg, 5 mg/kg, 10 mg/kg and 20 mg/kg (1/40^th^, 1/20^th^, 1/10^th^ and 1/5^th^ of LC_50_, respectively) for fourteen days. The results revealed that 22 metabolites (mainly fatty acids, sugars and amino acids) were shown significant responses in the exposed earthworms and these responses are dose dependent. It is proposed that mainly carbohydrate and fatty acids in neural system metabolism was disturbed. Overall, the results provided that metabolomics can be an effective tool to understand the effects of cypermethrin on the metabolic responses of earthworm *Metaphire posthuma*.

Cypermethrin ((RS)-alpha-cyano-3-phenoxybenzyl (1RS) cis-trans-3-(2, 2-dichlorovinyl)-2, 2-dimethyl-cycloproapane carboxylate) is one of the most widely used class-II synthetic pyrethroid for agricultural and domestic purposes globally^1^,^2^. The high photostability of cypermethrin than any other pyrethroid makes it very widely applicable as insecticide for agricultural applications. During the past 25 years, cypermethrin has become one of the dominant classes of insecticide, increasingly replacing carbamate and organophosphates in agriculture usage in Europe, Asia and North America^3^. Cypermethrin has been identified as one of the most important constituent pesticides associated with human health risks such as different neurological disorders, and Parkinson disease^4^,^5^. It readily crosses the blood brain barrier which in turn induces motor incoordination and finally modulates the levels of γ-aminobutyric acid (GABA) and sodium channels^6^,^7^. The main specific target of cypermethrin has been identified to be the sodium channels, which are kept open for long periods, causing prolonged sodium current to flow, which in turn, leads to hyper excitation of the nervous system^6^,^7^.

Pollution of the environment with pesticides is of great concern. Most of the pesticides strongly associate with organic matter in the soil which increases their residence time in the environment and subsequently increases the potential risk^8^,^9^,^10^. Despite of its beneficial roles, its repetitive, uncontrolled and liberal applications lead to unintended exposure to humans and animals, thereby increasing the risk of intoxication in non-target organisms and consequently higher ecotoxicological risk^11^. The greatest challenge is to measure soil health from organic contaminants. Soil quality and extent of toxicity due to contaminants is measured by finding the effect of these contaminants on earthworms, because earthworms are excellent model organism in terrestrial invertebrates, represent more than 60% of the soil total biomass^12^,^13^,^14^. Earthworms are ubiquitous in a vast range of soils and their well being is directly connected to the microbial community health and biodiversity^15^. Earthworms play an important role in soil macrofauna biomass and extremely play a crucial function in the turnover of soil organic matter and nutrients in natural and agricultural ecosystems and they have been described as soil engineers because they create biogenic structures with different physical, chemical and biological properties to surrounding soil aggregates^16^.

The relative abundances of earthworm species provide a gross indication of the health of the soil and it is expected that land use would affect earthworm metabolism as the populations rose or declined in response to changing soil health parameters. A biological approach to circumvent difficulties in studying soil biota is to use earthworms as an ecologically important group as indicators. It is a candidate bio-monitor organism for testing the chemical toxicity of soil^12^. Hence, it is an excellent and most widely used organism in eco-toxicology^12^,^17^. Understanding the biological responses of terrestrial invertebrates to these pesticides is of key importance for soil health risk assessment and subsequently to the environment^18^.

Most of biomarker tests employed to assess the toxicity on this model organism had limited ability to predict detrimental effects meaningful to risk assessment, namely, survival, growth, development, reproduction, toxic effects on some specific organ(s) and measurement of specific responses such as oxidative stress or specifically expressed genes and proteins to test for certain toxicities^19^,^20^. These are largely lacking of mechanistic knowledge concerning the linkages between molecular alterations and outcomes in the whole organism. To achieve a comprehensive understanding of the effects of toxicants and toxic mechanisms, a global analysis on the biological responses to these toxicant exposures and corresponding biomarkers should be performed at the molecular level. Omics tools offer substantial promise for the discovery of gene, protein or metabolite alterations indicative of mode of action of chemicals and improved understanding of the mechanistic action of prospective studies^21^. Endogenous metabolites are important factors in cellular regulatory and metabolic processes that respond to toxicological effects. Metabolomics, the untargeted analysis of endogenous metabolites from biological samples is clearly complementary to other omics approaches and have a special role in bridging the genotype-phenotype gap, since analysis of the metabolome can reflect the sum of all up-stream regulatory events, as well as direct inputs from external environment^22^. Metabolomics approach using mass spectrometry combined with computer-based pattern recognition methods allows simultaneous detection of hundreds of low molecular weight metabolites within a biological matrix, which has been proven to be a rapid and effective way of obtaining high qualitative and quantitative information on endogenous metabolites in whole organisms^23^,^24^. It is increasingly being used for the supervision of pathophysiologic processes and toxicity assessment, identification of target, cites of toxicity, evaluating the toxicity of chemical analytes and for the characterization of candidate biomarkers to toxicant exposure^21^,^25^,^26^,^27^,^28^. The uses of earthworm for metabolomic studies have been increasing gradually to identify the biomarkers of exposure to environmental contaminants such as polyaromatic hydrocarbons (pyrene, phenanthrene) different organic pollutants (DDT, endosulfan, polychlorinated biphenyls, carbofuran, and metal contaminants (copper, titanium dioxide)^29^,^30^,^31^,^32^,^33^,^34^,^35^,^36^,^37^.

Cypermethrin is a widely used pyrethroid insecticide and occupies fourth position globally in pest application. The usage is more than one million pounds per year in the US only as an active ingredient for agriculture necessities. However, despite its wide usage and knowledge on the toxicity of cypermethrin, surprisingly, no studies have been carried out till today on earthworms to evaluate the exposure and effects of this pyrethroid in metabolite level to identify biomarkers of exposure and toxicity mechanism of action. In considering these facts, an attempt has been made to identify the biochemical responses of cypermethrin on earthworm *Metaphire posthuma*. A non-targeted global metabolomic profiling was carried out using gas chromatography and mass spectrometry (GC-MS) combined with pattern recognition approaches to explore the metabolic perturbations on earthworm *Metaphire posthuma* to cypermethrin. *Metaphire posthuma* is an endogenous soil dwelling species, widely used for soil ecotoxicity studies and recently, we have explored and characterised this species for metabolomic studies^35^.

## Results

**Figure 1:**
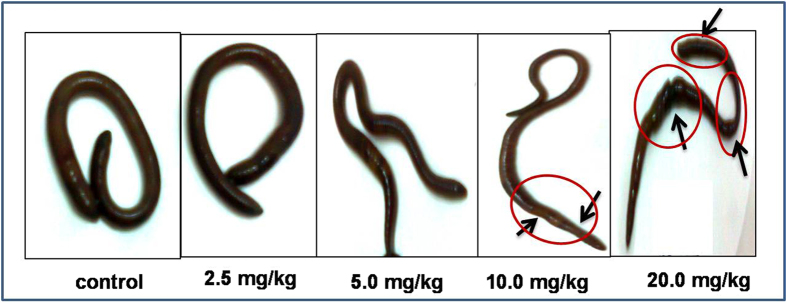
Physical changes appeared from the experimental worms of control and cypermethrin exposed at concentration of 2.5 mg/kg, 5.0 mg/kg, 10.0 mg/kg, and 20.0 mg/kg. Clear tissue damage is observed in at concentration of 10 mg/kg and 20 mg/kg indicated with arrows.

**Figure 2:**
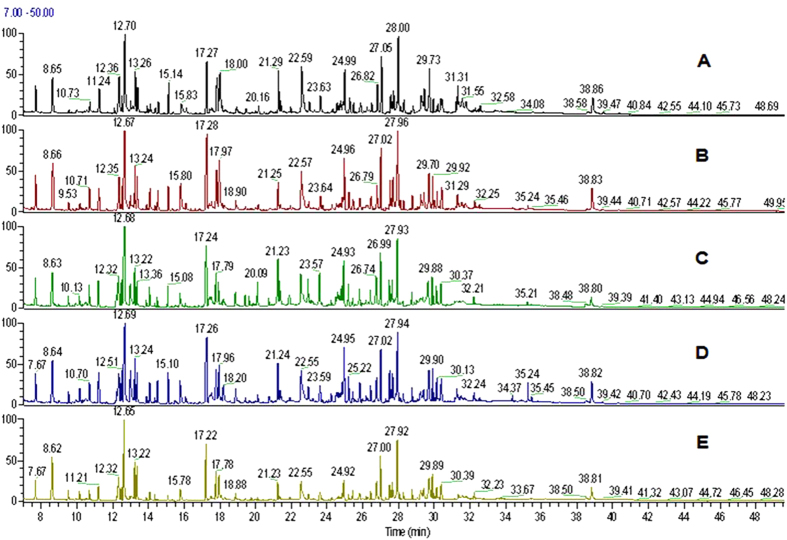
Typical GC-MS total ion current chromatogram (TIC) of earthworm metaphire posthuma tissue extracts obtained from (**A**) control and exposed cypermethrin concentration of (**B**) 2.5 mg/kg, (**C**) 5 mg/kg, (**D**) 10 mg/kg, (**E**) 20 mg/kg.

**Figure 3:**
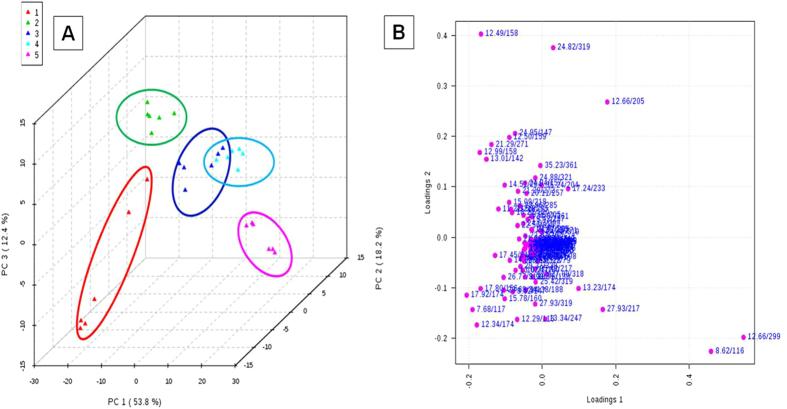
(**A**) Principal component analysis (PCA) scores plot of PC1 (first principal component) vs PC2 (second principal component) vs PC3 (third principal component) for *Metaphire posthuma* extracts showing the separation of (1) control worms, and exposed worms at concentrations of (2) 2.5 mg/kg, (3) 5 mg/kg, (4) 10 mg/kg, (5) 20 mg/kg. (**B**) Loadings plot for PC1 and PC2 showing the metabolites (rt/m/z) that were major contributors to the separation of groups observed in PCA score plot of earthworm *Metaphire posthuma* tissue extract.

**Figure 4:**
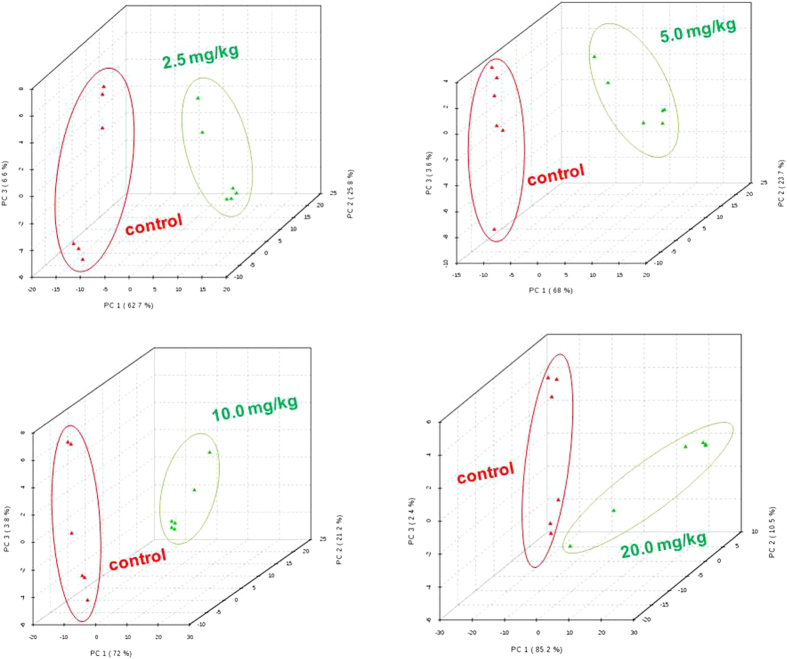
PCA scores plots of PC1 (first PCA component) versus PC2 (second PCA component) versus PC3 (third PCA component) for GC-MS spectra of the extract of the *Metaphire posthuma* tissue extracts showing the separation of control worms from exposed worms after cypermethrin exposures of (**A**) 2.5 mg/kg, (**B**) 5.0 mg/kg, (**C**) 10.0 mg/kg (**D**) 20.0 mg/kg for fourteen days.

**Figure 5:**
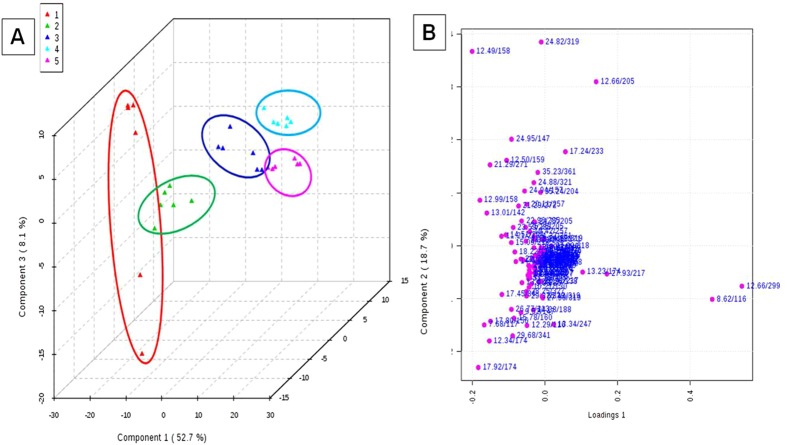
(**A**) Partial Least Square Discriminate analysis (PLS-DA) scores plot of first component vs second component vs third component for *Metaphire posthuma* extracts showing the separation of (1) control worms, from cypermethrin exposed worms of concentration of (2) 2.5 mg/kg, (3) 5 mg/kg, (4) 10 mg/kg, (5) 20 mg/kg. (**B**) Loadings plot for component 1 and component 2 showing the metabolites (rt/m/z) that were major contributors to the separation of groups observed in PCA score plot of earthworm *Metaphire posthuma* tissue extract.

**Figure 6:**
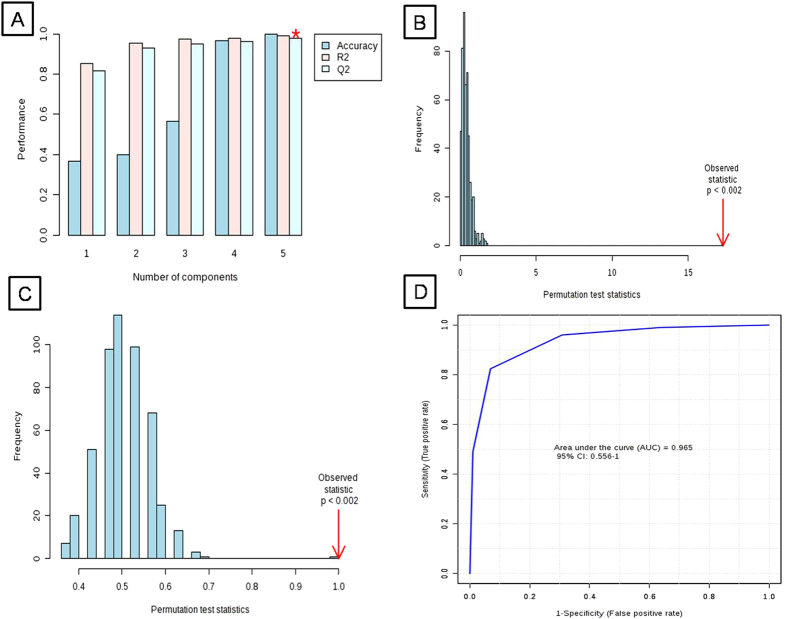
(**A**) Cross validation, (**B**,**C**) Permutation analysis of PLS-DA models derived from cypermethrin exposed and controls of earthworm *Metaphire posthuma* tissue extracts. Statistical validation of the PLS-DA by permutation analysis using 500 different model permutations. The goodness of fit and predictive capability of the original class assignments is much higher compared to ratios based on the permutation class assignments. (**D**) Predictive accuracy of the model discriminating cypermethrin exposed and healthy control earthworms summarised using ROC curve analysis. Area under the curve = 0.965.

**Figure 7:**
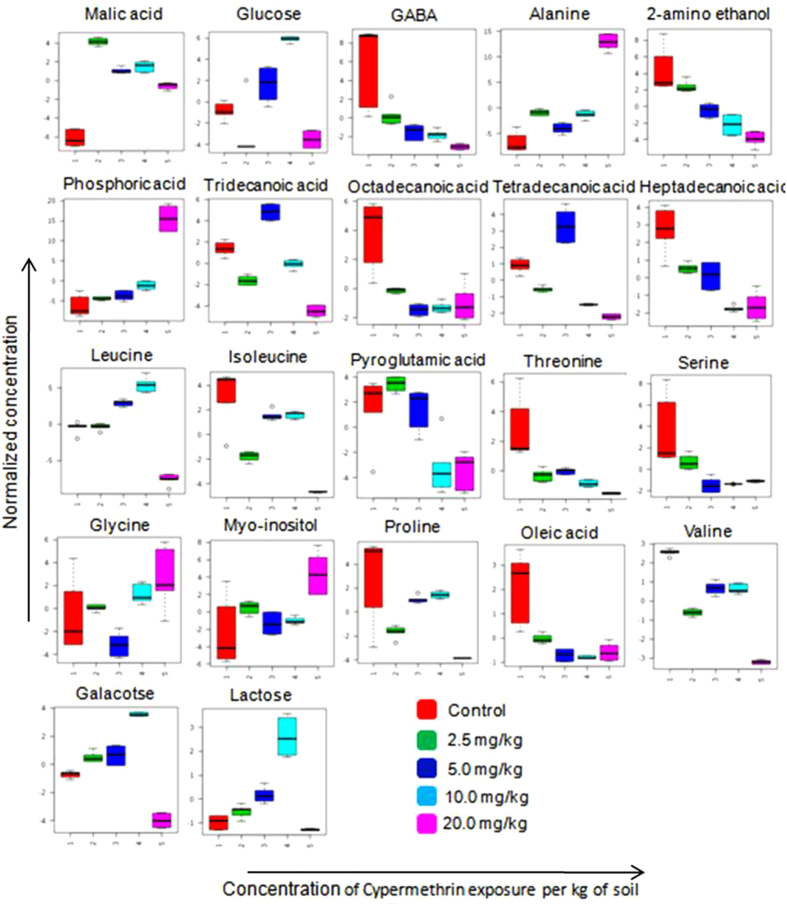
Earthworm metabolite responses to cypermethrin exposure 1) control group 2) concentration of 2.5 mg/kg exposed group 3) concentration of 5.0 mg/kg exposed group 4) concentration of 10.0 mg/kg exposed group 5) concentration of 20.0 mg/kg exposed group.

Earthworm’s mortality was not observed in any of the cypermethrin treatments after 14 days of exposure. All worms were survived in the experimental conditions and no worms were observed on the surface of the soil throughout the experimental period indicating that the test was robustly conducted^38^. The physical changes appeared on the worms were shown in Fig. 1. Metabolomic studies were carried out on earthworms at four sub-lethal concentrations, 20 mg/kg, 10 mg/kg, 5 mg/kg and 2.5 mg/kg corresponds to 1/5^th^ LC_50_, 1/10^th^ of LC_50_, 1/20^th^ of LC_50_ and 1/40^th^ of LC_50_^39^. The representative GC-MS total ion chromatograms (TICs) of earthworm extracts of control group, and cypermethrin exposed group of four different concentrations (2.5 mg/kg, 5.0 mg/kg, 10 mg/kg, 20 mg/kg) were shown in Fig. 2. Multivariate analysis was performed to reduce the data to low dimensional space, where discrimination of metabolic profiles between sample classes can be modeled. Initially unsupervised PCA was performed. The PCA results were displayed as scores plots indicating scatter of samples, which indicate similar metabolomic compositions when clustered together and compositionally different metabolomic compositions when dispersed. The unsupervised PCA model obtained from the GC-MS spectra of all samples revealed the general structure of the complete data set, in which two principle components cumulatively accounted for 72% of the total variance with PC1 explained 53.8% and PC2 explained 18.2% of the total variance respectively (Fig. 3). As presented in figure, five different clusters were identified in PCA scores plot. PCA scores plot showed that, there was significant separation (p < 0.05) between control and cypermethrin exposed earthworms along first principal component. Individual score plots were constructed to further characterize the dose trend in metabolic response using GC-MS data set across all four doses (2.5 mg/kg, 5.0 mg/kg, 10 mg/kg, 20 mg/kg). A clear, linear dose trend is evident in Fig. 4 with separation becoming more obvious the greater the dose. From individual score plots, it was clearly indicated that, the separation between control and cypermethrin exposed earthworms was greater, when the cypermethrin exposure concentration increases from 2.5 mg/kg (1/40^th^ of LC_50_) to 20 mg/kg (1/5^th^ of LC_50_). It was clearly depicted that the earthworm metabolic responses to cypermethrin exposure were concentration dependent. Supervised PLS-DA was further performed to find a small number of linear combinations of the original variables, which was predictive for the class membership and that, described most of the variability of the metabolic profiles of control and exposed samples. PLS-DA were performed to identify the metabolites that were responsible for the observed separation between scores of the control and cypermethrin exposed earthworms. From the scores plots, a clear clustering of individual samples with the group is evident (Fig. 5). Visual examination of PLS-DA score plots is not a reliable method for determining predictive power. Therefore internal cross validation was performed to find out the predictive accuracy and fit of the polynomial model. The cumulative values of PLS-DA model with R^2^X_cum_ = 0.543, R^2^Y_cum_ = 0.913, Q^2^Y_cum_ = 0.870 shows good fit of the model Fig. 6A. To assess the statistical significance of these apparently highly predictive multivariate models, permutation testing was conducted. The supervised models were further validated with 500 times permutation tests (Fig. 6). From the analysis of these distributions, the significance of the power of the optimal models to predict the chemical toxicities of cypermethrin exposed earthworms and control was determined to be p < 0.002 (Fig. 6B & 6C). Among all the differential variables selected according to the VIP values from the PLS-DA model (VIP>1), 22 metabolites, include long-chain fatty acids tridecanoic acid, tetradecanoic acid, heptadecanoic acid, octadecanoic acid, oleic acid, amino acids alanine, leucine, isoleucine, glycine, serine, threonine, proline, valine, amino acid derivative Pyroglutamic acid, messenger molecules inositol, GABA, sugars glucose, galactose, maltose, organic acid malic acid and other metabolites phosphoric acid, 2-Amino ethanol showed significant fluctuations in response to cypermethrin exposure (table 1). Classification performance of biomarkers was further evaluated with receiver operator characteristic curve (ROC) based on support vector machine (SVM). The area under the receiver operating characteristic curve (AUC) was used as a measure of classification performance. Model sensitivity and specificity were summarised using ROC curves for the models distinguishing cypermethrin exposed from healthy controls (AUROC = 0.965) (Fig. 6D). The normalized concentrations of metabolites intensities were further examined to determine the fluctuations in the metabolites of cypermethrin exposed samples relative to controls depicted in Fig. 7.

## Discussion

**Figure 8:**
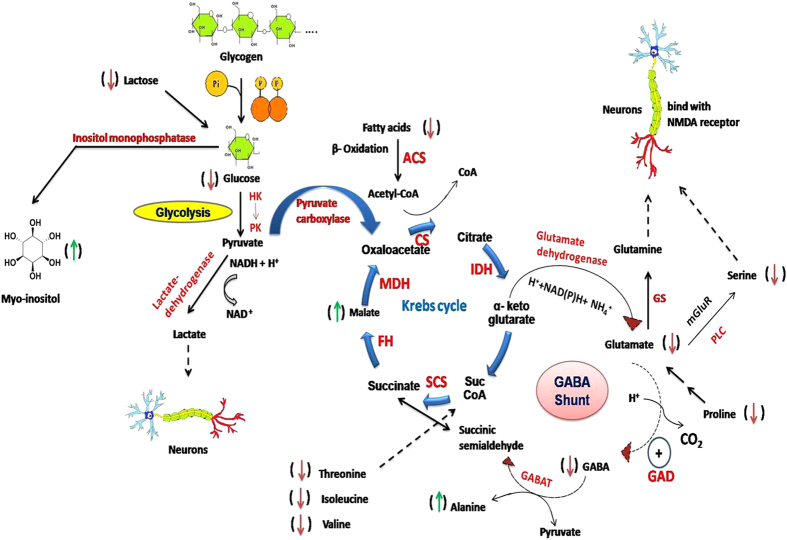
(Drawn by RCh, AKS & PP), Schematic representation of the affected pathways in earthworm *Metaphire posthuma* due to cypermethrin exposure. Enzymes: HK = Hexokinase, PK = Pyruvatekinase, CS = Citrate synthase, IDH = Isocitrate dehydrogenase, SCS = Succinyl coenzyme sinthetase, FH = Fumarate hydratase, MDH = Malate dehydrogenase, GABAT = GABA Aminotransferase, GAD = Glutamic acid decarboxylase, PLC = Phospholipase C, GS = Glutamine synthetase, ACS = Acyl CoA synthetase.

Metabolomics allows high-throughput analysis of low molecular weight metabolites in tissue that reflect the physiological status and biochemical metabolism of living systems and has the potential to evaluate toxicity and reveals the metabolism related toxic mechanisms. The present study reports the feasibility and effectiveness of utilizing GC-MS based metabolomic profiling to find out the metabolic fluctuations in earthworm *Metaphire posthuma* to the cypermethrin exposure that may provide further insights into toxicity mechanism of action. Earthworms were exposed to four different concentrations of cypermethrin based on their LC50 such as 2.5 mg/kg, 5 mg/kg, 10 mg/kg, 20 mg/kg of soil (corresponding 1/40^th^ of LC_50_, 1/20^th^ of LC_50_, 1/10^th^ of LC_50_, 1/5^th^ of LC_50_ respectively). After fourteen days exposure period metabolic profiles were screened with the use of GC-MS and multivariate analysis performed. Multivariate analysis clearly indicated that metabolic perturbations are occurring in cypermethrin exposed earthworms in comparison to control earthworms and these perturbations are concentration dependent. The schematic representation of main pathway was depicted in Fig. 8 and the summary of metabolic pathways were mentioned in table 2. Myo-inositol, a cyclic poly alcohol is the most abundant stereoisomer of inositol, plays an important role as secondary messenger in cell, in the form of inositol phosphates like phosphatidyl inositol cycle (PI-cycle)^40^,^41^. It is required for the synthesis of membrane inositol phospholipids and related signal transduction. In the present study, myo-inositol levels were increased (p < 0.05) in cypermethrin exposed earthworms relative to control indicating that phosphatidyl inositol phosphate metabolism was disturbed due to cypermethrin exposure. Myo-inositol is suggested to be a sensitive biomarker for earthworm exposure to cypermethrin.

GABA is a universal transmitter of junctional neuromuscular inhibition in all invertebrates^42^. GABA is a major inhibitory transmitter at both neuromuscular synapses and within the ganglia. GABA is basically inhibitory transmitter occupying the nerve receptor sites for anxiety and stress related messages. GABA is one of the most common targets of class II- pyrethroids, cypermethrin, which comes under this category and regulates the chloride channels^43^. Cypermethrin suppresses the open state of voltage gated chloride channels and inhibits GABA dependent uptake of chloride ions^44^,^45^. Cypermethrin induces neurotoxicity by modulating the levels of GABA. A previous study reveals that cypermethrin antagonizes GABA and causes the decrease in GABA levels^44^,^45^. In the present study decrease in GABA levels (p < 0.05) was observed. It is suggested to be GABA is a sensitive biomarker to cypermethrin exposure in earthworms. Cypermethrin mediated neurotoxicity may be due to its ability to inhibit the activity of acetyl cholinesterase in the brain, leading to decreased cholinergic transmission and consequent accumulation of the neurotransmitter acetylcholine resulting in the termination of nerve impulses.

Glucose is a primary carbon energy fuel for central nervous system^46^. Under the conditions of stress, utilization of glucose levels may occur due to high energy requirements of the brain. These glucose levels can be obtained from the depletion of reserve glycogen levels in the brain. In the present study decrease in glucose levels were observed in earthworms exposed to cypermethrin. In addition to glucose other carbohydrates like galactose and lactose were down regulated in cypermethrin exposed worms in comparison to control worms. Our findings supported by the previous literature that clearly indicates that cypermethrin known to induce alterations in carbohydrate metabolism. Reddy *et al*. (1991a) has demonstrated the carbohydrate metabolic diversions in cockroach, *Periplaneta Americana*, to counteract cypermethrin toxicity^47^. Similar type of observations has been reported in fish *Tilapia mossambica* under cypermethrin stress^48^. Our findings suggested that glucose, galactose and lactose are sensitive biomarkers for earthworms exposure to cypermethrin.

Long chain fatty acids are important constituents of cell membranes, particularly in the central nervous system (CNS)^49^. The incorporation of the long chain fatty acids in nerve cell membranes of the brain is one of the processes of perinatal development that contributes to the functional maturation of central nervous system. Normally the CNS relies primarily on glucose as a major energy source. Under conditions of metabolic stress, immune cells have the ability to utilize next alternative substrates such as fatty acids^50^,^51^. The energy demand for the CNS system is fulfilled by fatty acid metabolism (β-oxidation). In the current study, we observed that the depletion (p < 0.05) of fatty acids, tridecanoic acid, tetradecanoic acid, octadecanoic acid, heptadecanoic acid, dodecanoic acid and oleic acid are occurring in cypermethrin exposed earthworms relative to control. It indicates that, the energy requirement for CNS system by uptake of fatty acids is more. The more energy requirement for CNS system is may be due to stress caused by cypermethrin.

Pyroglutamic acid is a cyclic form of Glutamic acid. In central nervous system, it serves as an excitatory neurotransmitter. Glutamate as a neuro mediator undergoes oxidative degradation in neurons and in astroglia under the conditions of stress^52^. In the present study decrease in glutamate levels were observed in cypermethrin exposed worms in comparison to control worms suggesting that Pyroglutamic acid is a sensitive biomarker for cypermethrin exposure.

D-serine is a physiological co-agonist of N-methyl D-aspartate (NMDA) type of glutamate receptor- a key excitatory neurotransmitter receptor in the brain^53^. D-serine binds with high affinity to a co-agonist site on the NMDA receptor and, along with glutamate, mediates several important physiological processes, including NMDA receptor transmission, synaptic plasticity and neurotoxicity^54^. In the present study decrease (p < 0.05) in the serine levels were observed in earthworms exposed to cypermethrin relative to controls. It may be due to neurological dysfunction caused by cypermethrin.

Alanine considered as a universal indicator under stress conditions in various different organisms have been reported^32^,^55^. The increasing levels of alanine (at p < 0.05) in cypermethrin exposed earthworms compared to control, indicates that alanine may be a very sensitive indicator to cypermethrin exposure. It may be due to increase in gluconeogenesis, since alanine is one of its major substrate^56^. In case of mammalian kidneys, increase in alanine levels directly indicates stimulates of stress protein production. Our findings are supported by the previous reported study of the effect of cypermethrin on fish tilapia Mozambique, where increases in alanine levels were observed in fish exposed to cypermethrin^57^.

Malate, an important intermediate of TCA cycle and the enzyme malate dehydrogenase (MDH) catalyzes the intercoversion of oxaloacetate to malate for energy requirements. In the present study, elevated levels of malate (p < 0.05) are observed in earthworms exposed to cypermethrin, in comparison to control earthworms. It may be due to high energy requirement of earthworms under stress conditions caused by cypermethrin exposure. It is supported by the earlier studies that malate dehydrogenated levels including both cytosol MDH and mitochondria MDH were altered in earhworms from different ecophysiological category (including *Metaphire posthuma*) after their exposure to cypermethrin^58^,^59^.

Valine, leucine and isoleucine are three essential branched chain amino acids plays important role in energy and muscle metabolism^60^,^61^. The decrease in valine is marked by the neurological defects in the brain. The decrease in isoleucine may be due to the stress occurred in muscle metabolism. Our findings are supported by previous studies of cypermethrin effect on earthworm metaphire posthuma that decline in protein content was observed. It is further supported by the previously reported study of the effect of cypermethrin on total protein in muscle and liver of freshwater fish *Channa striatus*^62^.

The work presented here is the first metabolomics study, describes the utilization of GC-MS based non-targeted metabolomics approach to identify the perturbations in metabolic profile of earthworm after their exposure to cypermethrin. Multivariate pattern recognition analysis showed clear molecular group responses to cypermethrin at metabolic level and concentration dependent metabolic perturbations were observed. The integrated data set indicated clear alteration of neuronal metabolism as a result of sub-lethal cypermethrin exposure, with an increased switch to glycogen consumption and fatty acid consumption, presumably as a result of cypermethrin interfering with sodium channels and reducing the neurotransmitter GABA causing stress. The work demonstrated that metabolomics has great potential to emerge as a powerful ecotoxicological tool for determining the mechanism of action to better understand the toxicological effects of environmental pollutants.

## Methods

### Chemicals and Reagents

All chemicals used were of analytical grade unless otherwise stated. Methoxyamine hydrochloride, cypermethrin and N-methyl-N-trimethylsilyl trifluoroacetamide (MSTFA) and all standards of amino acids, sugars and organic acids were procured from Sigma-Aldrich (St. Louis, MO, USA). Methanol was obtained from Sigma-Aldrich (St. Louis, MO, USA). The ultra pure water was prepared by RiOsTM water purification system (Millipore, Billerica, MA, USA). IMECO ULTRA SONICS (Bombay, India) was used as sonicator. Heto GD-2 maxi dry plus was used as a lyophilizer.

### Experimental design

The experimental setup was carried out as per previously reported procedures, is summarized briefly below^33^,^36^,^38^,^63^. The test worms (*Metaphire posthuma*, weighing > 1.10 g) were collected from culture and placed in soil to acclimatize for a seven days in a BOD incubator at 20 ± 1 °C. The constituents of the soil were measured and found to be nitrogen 64 mg/kg, phosphorous 8.3 mg/kg, potassium 28 mg/kg, magnesium 36 mg/kg, sand 87% and pH 7.1. One kilogram (dry weight) of soil mix was placed into 2 litre jars and spiked with concentrations of 0, 2.5, 5.0, 10, and 20 mg/kg of cypermethrin in an equal volume of acetone. Soils were then vented for 72 h to remove all the solvent, wetted to 60% of water holding capacity and then left to stabilize for 24 h prior to addition of the worms. Six worms were added as a batch to each jar and the experiment was conducted for 14 days at 20 ± 1 °C in a 16:8 light: dark regime as per OECD guidelines^38^. After 14 days, the surviving worms were retrieved from the soil and weighed. Worms were then kept on the moist filter papers for 24 h to allow soil to be egested from the gut. Individuals were then snap-frozen into liquid nitrogen and stored at −80 °C.

### Metabolite extraction and derivatization

Metabolite extraction and derivatization were carried out as per previously reported procedure with little modification^31^. Briefly, initially earthworms were flash-frozen in liquid nitrogen, lyophilized and kept under frozen conditions until extraction. The lyophilized earthworms were homogenized in centrifuge tube using a spatula. To the homogenized earthworm tissue, 1 ml of 80% methanol was added and vortexed for 2 min followed by 5 min sonication. Then the tissue mixture was centrifuged for 10 min at 10,000 rpm. All the extractions were carried out in triplicate with 80% methanol under ice-cold conditions. The aliquot was transferred into 2 ml of eppendorf tubes for lyophilyzation. To the dried samples 90 μl of O-methoxyamine hydrochloride solution in anhydrous pyridine was added, mixed vigorously for 1 min, and incubated at 65 °C for 30 min in a heating block. Subsequently, 140 μl of MSTFA was added and the extracts were incubated at 60 °C for 60 min. Samples were then made up to a volume of 800 μl with hexane prior to GC-MS analysis.

### GC-MS instrumentation and data acquisition

Metabolite profiling was performed on Trace GC ultra (Thermo Scientific, FL, USA) coupled to TSQ Quantum XLS mass spectrometer (Thermo Scientific, FL, USA). TG-5MS fused-silica capillary column (30 m × 250 μm i.d; Thermo Scientific), chemically bonded with a 5% phenyl 95% methyl polysiloxane cross linked stationary phase (0.25 μm film thickness) was utilized to separate the derivatives. The injector temperature was set at 250 °C. Helium, the carrier gas, was maintained at a constant flow rate of 1.1 ml/min during the analysis. The column temperature was initially kept at 65 °C for 2 min, then ramped to 230 °C at a rate of 6 °C/min and then finally increased to 290 °C at a rate of 10 °C/min, where it was held for 20 min. The interface temperature and ion source were set at 290 °C and 220 °C respectively. Electron ionization (EI^+^) mode was used for mass detection with electron energy of 70 eV. Mass spectra were acquired with a scan range of m/z 45-800. Solvent delay was set at 7 min. The sample volume of 1 μl was injected for GC-MS analysis.

### Data pre-processing and multivariate pattern recognition

Automated peak detection, retention time alignment and peak matching were carried out on CDF files using XC-MS software (http://masspec.scripps.edu/xcms/xcms.php)^64^. The data were arranged in a three-dimensional matrix consisting of an arbitrary peak index (Rt- *m/z* pair), sample names, and peak area. Total area normalization was performed in order to reduce the systematic biases within the experiment^65^,^66^. Scaled data (Pareto scaled) were used for multivariate analysis to remove the offsets and adjust the importance of high and low abundance metabolites to an equal level. Multivariate statistical analysis was performed by using Metaboanalyst (www.metaboanalyst.ca)^67^. Principal component analysis (PCA) was performed to explore the clustering behaviour of metabolites. To identify the differential metabolites that account for the separation between groups, partial least square discriminate analysis (PLS-DA) was applied. PLS-DA model was validated using the leave one out cross validation method^68^. Quality of model is assessed on R^2^ and Q^2^ scores. In addition to cross validation, model validation was also performed by 500 times permutation tests^68^,^69^,^70^. Metabolites with variable importance in projection (VIP) values of greater than 1 were identified as potential marker metabolites^70^. AMDIS software (Automated Mass Spectral Deconvolution and Identification System, version 2.0, NIST, USA) was used to identify metabolites in chromatograms and the mass spectra of all detected compounds were compared with spectra in the NIST library (NIST, version 2.0, USA) or Golm metabolome database^71^. Further, metabolites such as amino acids and carbohydrates were identified by comparison to mass spectra and retention times of an *in-house* reference mass spectral database, that were generated with authentic standard compounds under the similar data acquisition parameters. Univariate analysis was performed to these metabolites by applying unpaired *t*-test with a significance value (*p*) less than 0.05. Classification performance of biomarkers was further evaluated with receiver operator characteristic curve (ROC) based on support vector machine (SVM)^72^. Classification performance was measured using area under receiver operating characteristic curve (AUC).

### Pathway analysis

Summary of metabolic pathways affected due to cypermthrin exposure was performed using MetPA^73^,^74^. It is based on the KEGG metabolic pathways as the build up knowledge to identify biological pathways. Default settings Hypergonemtetric test was used for over representation analysis and out-degree centrality used for the pathway topology analysis. All the affected pathways given by MetPA are taken into consideration without setting any specific threshold^72^.

## Additional Information

**How to cite this article**: Ch, R. *et al*. Identifying the metabolic perturbations in earthworm induced by cypermethrin using gas chromatography-mass spectrometry based metabolomics. *Sci. Rep*. **5**, 15674; doi: 10.1038/srep15674 (2015).

## Figures and Tables

**Table 1:** Differential metabolites accountable for the discrimination between Cypermethrin exposed and control earthworms.

ChEBI ID	Metabolite	Retention timein min	m/z ofanalyzedfragment	VIPscore	*p*-value	%Probability as perNIST library
CHEBI:6650	Malic acid^a^	17.24	233	3.5351	3.5976E-21	76
CHEBI:17234	Glucose^a^	24.82	319	2.9978	1.4334E-7	80
CHEBI:16865	GABA^a^	17.92	174	2.6892	2.2749E-7	95
CHEBI:16977	Alanine^a^	8.62	116	2.6865	5.3988E-19	82
CHEBI:16000	2-Amino ethanol	12.34	174	1.9224	1.1084E-9	84
CHEBI:26078	Phosphoric acid	12.66	299	1.8769	2.4055E-15	79
CHEBI:45919	Tridecanoic acid^a^	21.29	271	1.7915	1.0114E-18	96
CHEBI:28842	Octadecanoic acid^a^	29.68	341	1.7256	9.1941E-8	97
CHEBI:28875	Tetradecanoic acid^a^	23.59	285	1.6296	1.5094E-15	98
CHEBI:32365	Heptadecanoic acid^a^	26.77	313	1.6275	8.3413E-10	98
CHEBI:15603	Leucine^a^	12.49	158	1.6092	1.4199E-20	82
CHEBI:17191	Isoleucine^a^	12.99	158	1.2819	3.1524E-12	75
CHEBI:18183	Pyroglutamic acid	17.80	156	1.4898	5.4772E-7	94
CHEBI:16857	Threonine^a^	13.02	117	1.4005	7.5697E-7	97
CHEBI:17115	Serine^a^	12.29	116	1.377	2.3006E-5	98
CHEBI:15428	Glycine^a^	13.23	174	1.358	2.8989E-4	90
CHEBI:17268	Myoinositol^a^	27.00	318	1.2075	0.0023104	83
CHEBI:17203	Proline^a^	13.01	142	1.1408	5.7539E-7	79
CHEBI:16196	Oleic acid^a^	29.37	339	1.0632	1.0487E-7	86
CHEBI:16414	Valine^a^	11.21	218	1.1618	6.2002E-24	81
CHEBI:28260	Galactose^a^	24.95	147	1.5966	6.532E-19	80
CHEBI:17306	Lactose^a^	35.23	361	1.2361	2.0949E-13	95

^a^Identification was unequivocally confirmed by comparison of retention times and spectral data to the corresponding pure standards.

**Table 2:** Summary of affected metabolic pathways in earthworm, *Metaphire posthuma* due to cypermethrin exposure.

Pathway Name	Hits	Metabolites	Impact
Valine, leucine and isoleucine biosynthesis	4	Threonine, Leucine, Isoleucine, valine	0.625
Glycine, serine and threonine metabolism	3	Threonine, Serine, Glycine	0.42307
Ascorbate and aldarate metabolism	1	Myo-inositol	0.25
Galactose metabolism	3	Lactose, Glucose, Galactose	0.23077
Cyanoamino acid metabolism	3	Glycine, Serine, Alanine	0.2
Pantothenate and CoA biosynthesis	1	Valine	0.2
Aminoacyl-tRNA biosynthesis	7	Glycine, Serine, Vanine, Isoleucine, Leucine, Threonine, Proline	0.17776
Methane metabolism	2	Glycine, Serine	0.16667
Glyoxylate and dicarboxylate metabolism	1	Malic acid	0.14286
Inositol phosphate metabolism	1	Myo-inositol	0.11111
Pyruvate metabolism	1	Malic acid	0.09091
Arginine and proline metabolism	2	Proline, GABA	0.08333
Citrate cycle (TCA cycle)	1	Malic acid	0.06897
Glutathione metabolism	2	Pyroglutamic acid, Glycine	0.06896
Sphingolipid metabolism	1	Serine	0.06667
Butanoate metabolism	1	GABA	0.0625
Valine, leucine and isoleucine degradation	3	Valine, Leucine, Isoleucine	0.06249
Starch and sucrose metabolism	1	Glucose	0.05
Alanine, aspartate and glutamate metabolism	1	GABA	0.05
Cysteine and methionine metabolism	1	Serine	0.04348
Amino sugar and nucleotide sugar metabolism	1	Galactose	0.03571
Fatty acid metabolism	1	Hexadecanoic acid	0.02083
Nitrogen metabolism	1	Glycine	0.0
Fatty acid biosynthesis	2	Hexadecanoic acid, Tetradecanoic acid	0.0
Glycerophospholipid metabolism	1	Ethanol amine	0.0
